# Supplementary material for: The eukaryotic replisome tolerates leading‐strand base damage by replicase switching
Source: EMBO J. 2021 Feb 8;40(5):e107037. doi: 10.15252/embj.2020107037 (PMC7917549; doi:10.15252/embj.2020107037)
Supplement: Supplementary file 1 — Appendix [file EMBJ-40-e107037-s001.docx]

**APPENDIX FOR**

**The eukaryotic replisome tolerates leading-strand base damage by replicase switching**

Thomas A. Guilliam^1^ and Joseph T.P. Yeeles^1,2,*^

**Table of Contents:**

**- Supplementary Table S1.** Key resources and reagents.

**- Supplementary Table S2.** Oligonucleotides used in this study.

**- Supplementary Table S3.** Yeast Strains originating in this study and used for protein expression.

**- Supplementary Table S4.** Plasmids used to generate yeast strains originating in this study.

**- Supplementary Table S5.** Protocol references, tags, purification methods, and final buffer composition for purified proteins used in replication assays.

| REAGENT or RESOURCE | SOURCE | IDENTIFIER |
| --- | --- | --- |
| Bacterial and Virus Strains | | |
| 5-alpha Competent *E. coli* (High Efficiency) | New England Biolabs | Cat# C2987H |
| *Escherichia coli*: Rosetta 2(DE3) strain: F^-^ *ompT hsdS*_B_(r_B_^-^ m_B_^-^) *gal dcm* (DE3) pRARE2 (Cam^R^) | Novagen | Cat# 71400 |
| Chemicals, Peptides, and Recombinant Proteins | | |
| 3xFLAG peptide | Sigma-Aldrich | Cat# F4799 |
| Anti-FLAG M2 affinity gel | Sigma-Aldrich | Cat# A2220 |
| Calmodulin-Sepharose 4B | GE Healthcare | Cat# 17-0529-01 |
| Calmodulin Affinity Resin | Agilent | Cat# 214303 |
| Glutathione-Sepharose 4B | GE Healthcare | Cat# 17-0756-01 |
| Talon metal affinity resin | Clontech | Cat# 635502 |
| Bio-Gel HT Hydroxyapatite | Bio-Rad | Cat# 1300150 |
| Ni-NTA Agarose | QIAGEN | Cat# 30210 |
| IgG-Sepharose Fast Flow | GE Healthcare | Cat# 17-0969-01 |
| cOmplete, EDTA-free | Roche | Cat# 5056489001 |
| Microspin G-50 columns | GE Healthcare | Cat# GE27-5330-02 |
| Anti-PCNA antibody | Abcam | Ab70472 |
| Goat anti-Mouse IgG HRP antibody | ThermoFisher | Cat# A16078 |
| Ubiquitin | Abcam | Ab189715 |
| Cdt1-Mcm2-7 | Coster et al., 2014 | N/A |
| ORC | Frigola et al., 2013 | N/A |
| Cdc6 | Frigola et al., 2013 | N/A |
| DDK | On et al., 2014 | N/A |
| Sld3/7 | Yeeles et al., 2015 | N/A |
| Cdc45 | Yeeles et al., 2015 | N/A |
| Dpb11 | Yeeles et al., 2015 | N/A |
| Sld2 | Yeeles et al., 2015 | N/A |
| GINS | Yeeles et al., 2015 | N/A |
| Pol ε | Yeeles et al., 2015 | N/A |
| S-CDK | Yeeles et al., 2015 | N/A |
| Mcm10 | Yeeles et al., 2015 | N/A |
| Pol α | Yeeles et al., 2015 | N/A |
| RPA | (Devbhandari et al., 2017) | N/A |
| Ctf4 | Yeeles et al., 2015 | N/A |
| Topo I | Yeeles et al., 2017 | N/A |
| Mrc1 | Yeeles et al., 2017 | N/A |
| Csm3/Tof1 | Yeeles et al., 2017 | N/A |
| RFC | Yeeles et al., 2017 | N/A |
| PCNA | Yeeles et al., 2017 | N/A |
| Pol δ | Yeeles et al., 2017 | N/A |
| Pol δ^cat^ | Aria and Yeeles, 2019 | N/A |
| Pol η | Guilliam and Yeeles, 2020 | N/A |
| Rad6/Rad18 | Guilliam and Yeeles, 2020 | N/A |
| Uba1 | Guilliam and Yeeles, 2020 | N/A |
| Pol ζ | This study | N/A |
| Rev1 | This study | N/A |
| Pol ε^exo-^ | This study | N/A |
| Experimental Models: Organisms/Strains | | |
| yAM33 (Cdt1-Mcm2-7 purification) | Coster et al., 2014 | N/A |
| ySD-ORC (ORC purification) | Frigola et al., 2013 | N/A |
| ySDK8 (DDK purification) | On et al., 2014 | N/A |
| yTD6 (Sld3/7 purification) | Yeeles et al., 2015 | N/A |
| yTD8 (Sld2 purification) | Yeeles et al., 2015 | N/A |
| yJY13 (Cdc45 purification) | Yeeles et al., 2015 | N/A |
| yJY26 (Dpb11 purification) | Yeeles et al., 2015 | N/A |
| yAJ2 (Pol ε purification) | Yeeles et al., 2015 | N/A |
| yAE37 (S-CDK purification) | Yeeles et al., 2015 | N/A |
| yAE40 (Ctf4 purification) | Yeeles et al., 2015 | N/A |
| yJY23 (Pol α purification) | Yeeles et al., 2015 | N/A |
| yAE34 (Pol δ purification) | Yeeles et al., 2017 | N/A |
| yAE41 (RFC purification) | Yeeles et al., 2017 | N/A |
| yAE42 (Topo I purification) | Yeeles et al., 2017 | N/A |
| yAE48 (Csm3/Tof1 purification) | Yeeles et al., 2017 | N/A |
| yJY32 (Mrc1 purification) | Yeeles et al., 2017 | N/A |
| yVA28 (Pol δ^cat^ purification) | Aria and Yeeles, 2019 | N/A |
| yTG1 (Pol η purification) | Guilliam and Yeeles, 2020 | N/A |
| yTG3 (Rad6–Rad18 purification) | Guilliam and Yeeles, 2020 | N/A |
| yTG4 (Uba1 purification) | Guilliam and Yeeles, 2020 | N/A |
| yTG2 (Rev1 purification) | This study | N/A |
| yTG7 (Pol ζ purification) | This study | N/A |
| yTG11 (Pol δ purification, *ΔREV3*) | This study | N/A |
| yJY108 (Pol ε^exo-^ purification) | This study | N/A |
| Oligonucleotides | | |
| See Table S1 for all DNA oligonucleotides used in this study |  |  |
| Recombinant DNA | | |
| ZN3: ARS306 replication template | Taylor and Yeeles, 2018 | N/A |
| ZN3 Tg1: BspQI ARS306 replication template | This study | N/A |
| pJM126 (RPA purification) | Addgene | #49339 |
| pAM3 (Cdc6 purification) | Frigola et al., 2013 | N/A |
| vJY19 (PCNA purification) | Yeeles et al., 2017 | N/A |
| pJFDJ5 (GINS purification) | Yeeles et al., 2015 | N/A |
| pET28a-Mcm10 (Mcm10 purification) | Yeeles et al., 2015 | N/A |
| Software and Algorithms | | |
| ImageJ | National Institute of Health | <https://imagej.nih.gov/ij/> |

**Supplementary Table S1. Key resources and reagents.**

| **Oligonucleotides (5ʹ-3ʹ)** | **SOURCE** | **IDENTIFIER** |
| --- | --- | --- |
| Nt. BspQI removal FWD: GATCATCCGCTTCCTCGCTCACTGACTCG | This study | N/A |
| Nt. BspQI removal REV: GGAAGCGGATGATCGCCCAATACGCAAACC | This study | N/A |
| Nt. BspQI addition FWD: GTCGCTCTTCTCGAGGTTTGCCATATCGGTCCG | This study | N/A |
| Nt. BspQI addition REV:  CTCGAGAAGAGCGACAATGGAACCGCTATCAATACC | This study | N/A |
| Integrated CPD oligonucleotide:  TCAGCAC-/CPD/-AAGTCC | Taylor and Yeeles, 2018 | N/A |
| Integrated Tg oligonucleotide:  TCAGCACT-/Tg/-AAGTCC | This study | N/A |
| Integrated 8oxoG oligonucleotide:  TCAGCACT-/8oxoG/-AAGTCC | This study | N/A |
| For artificial restart assays: 21 nt re-priming: GGTTGGTACTGCGG | Taylor and Yeeles, 2018 | N/A |
| For artificial restart assays: 21 nt scrambled: GGTGATCGTTCGGG | Taylor and Yeeles, 2018 | N/A |
| For artificial restart assays: 265 nt re-priming: CTGGTTTCCGCCGT | Taylor and Yeeles, 2018 | N/A |
| For primer extension assays: Tg_50: TTAAATTAAGTGTTAACTCTgACTCAAGTGGTGAGTTGGCACGTGTACAAG | This study | N/A |
| For primer extension assays: Cy3_20P:  Cy3-CTTGTACACGTGCCAACTCA | This study | N/A |
| For primer extension assays: Cy3_30P:  Cy3-CTTGTACACGTGCCAACTCACCACTTGAGT | This study | N/A |
| Competitor DNA:  CTTGTACACGTGCCAACTCACCACTTGAGTAGAGTTAACACTTAATTTAA | This study | N/A |
| Primer for ZN3 SwaI sequencing ladder: AAATAAAACCAATCCCGAG | This study | N/A |
| Byp_Fid_FWD: GTTTCTTAAGCTTCTCCTAAGGACAGCACGAATC | This study | N/A |
| Byp_Fid_REV:  GTTTCTTGGTACCGTCGACTTATATCCTGCTCTTAAAGG | This study | N/A |
| For deletion of *REV3* FWD: ATTTGAGTCAATACAAAACTACAAGTTGTGGCGAAATAAAATGTTTGGAACGTACGCTGCAGGTCGAC | This study | N/A |
| For deletion of *REV3* REV: ATAGAAACAAATAACTACTCATCATTTTGCGAGACATATCTGTGTCTAGAATCGATGAATTCGAGCTCG | This study | N/A |

**Supplementary Table S2. Oligonucleotides used in this study.**

| **Strain** | **Genotype** |
| --- | --- |
| yTG2 | *MATα ade2-1 ura3-1 his3-11,15 trp1-1 leu2-3,112 can1-100*  *bar1::Hyg*  *pep4::KanMX*  *his3::HIS3pRS303-CBP-Rev1* |
| yTG7 | *MATα ade2-1 ura3-1 his3-11,15 trp1-1 leu2-3,112 can1-100*  *bar1::Hyg*  *pep4::KanMX*  *trp1::TRP1pRS304-2xFLAG-REV3+REV7*  *ura3::URA3pRS306-POL31+POL32-CBP* |
| yTG11 | *MATα ade2-1 ura3-1 his3-11,15 trp1-1 leu2-3,112 can1-100*  *bar1::Hyg*  *pep4::KanMX*  *ura3::URA3pRS306-POL31+POL3*  *his3::HIS3pRS303-POL32-CBP+Gal4*  *REV3Δ::natMX6* |
| yJY108 | *MATa ade2-1 ura3-1 his3-11,15 trp1-1 leu2-3,112 can1-100*  *bar1::Hyg*  *pep4::KanMX*  *POL2-3XFLAG-NatNT2*  *ura3::Ura3pRS306Gal1-10 Dpb2+Dpb3*  *trp1::TRP1pRS304Dpb4-CBP, Pol2-D290A, E292A (exo-)* |
|  |  |

**Supplementary table S3.** **Yeast Strains originating in this study and used for protein expression.**

| **Plasmid** | **Insert** | **Plasmid construction** |
| --- | --- | --- |
| pRS303/CBP-Rev1-Gal-Gal4 | CBP-Rev1 | Synthetic construct cloned 5ʹ- SgrAI, 3ʹ- NotI |
| pRS304/ 2xFLAG-Rev3+Rev7 | 2xFLAG-Rev3  Rev7 | Synthetic construct cloned 5ʹ- SgrAI, 3ʹ- NotI  Synthetic construct cloned 5ʹ- AscI, 3ʹ- XhoI |
| pRS306/Pol3-CBP+Pol31 | Pol32-CBP | Pol3 removed from pRS306/Pol3- Gal-Pol31  and Pol32 sub-cloned in from pRS303/Pol32- CBP-Gal-Gal4 by 5ʹ- SgrAI, 3ʹ- NotI |
| *pRS304Dpb4-CBP, Pol2-D290A, E292A* (vJY108) | *pRS304Dpb4-CBP, Pol2-D290A, E292A* | Site directed mutagenesis and subcloning of mutant Pol2 gene 5ʹ- AscI, 3ʹ- XhoI |

**Supplementary Table S4. Plasmids used to generate yeast strains originating in this study.**

| **Protein** | **Tag** | **Purified exactly as in** | **Purification steps** |  | **Final buffer composition** |
| --- | --- | --- | --- | --- | --- |
| Cdc45 | Internal 2xFLAG tag |  | Anti-FLAG M2 Agarose  Bio-Gel HT Hydroxyapatite |  | 25 mM HEPES-KOH pH 7.6, 10% glycerol, 1 mM EDTA, 1 mM DTT, 300mM KOAc |
| Cdc6 | N-terminal GST cleavable tag |  | Glutathione Sepharose 4B  Bio-Gel HT Hydroxyapatite |  | 50 mM K_2_HPO_4_/KH_2_PO_4_ pH 7.5, 5 mM MgCl_2_, 1% Triton X-100, 1 mM DTT 400 mM KOAc, 15% glycerol |
| Cdt1-Mcm2-7 | N-terminal CBP cleavable tag on Mcm3 | Coster et al., 2014 | Calmodulin-Sepharose 4B  Superdex 200 |  | 45 mM HEPES-KOH pH 7.6, 100 mM KOAc, 5 mM MgOAc, 0.02% NP40, 10% glycerol |
| Ctf4 | N-terminal CBP tag |  | Calmodulin-Sepharose 4B  MonoQ  Superdex 200 |  | 25 mM Tris-HCl pH 7.2, 10% glycerol, 1 mM DTT, 75 mM NaCl, 1 mM EDTA |
| DDK | CBP tag on Dbf4 |  | Calmodulin-Sepharose 4B  Lambda phosphatase dephosphorylation  Superdex 200 |  | 25 mM Hepes-KOH pH 7.6, 0.1 mM EGTA, 0.1 mM EDTA, 0.02% NP-40, 10% glycerol, 100 mM K-Glutamate, 2 mM β-mercaptoethanol |
| Dpb11 | C-terminal 3xFLAG tag | (Different storage buffer) | Anti-FLAG M2 Agarose  MonoS |  | 25 mM HEPES-KOH pH 7.6, 10% glycerol, 0.02% (v/v) NP-40-S, 1 mM EDTA, 1 mM DTT, 300mM KOAc |
| GINS | N-terminal His tag on Psf3 |  | Ni-NTA Agarose  MonoQ  Superdex 200 |  | 25 mM HEPES-KOH pH 7.6, 10% glycerol, 0.02% (v/v) NP-40-S, 1 mM EDTA, 1 mM DTT, 200mM KOAc |
| Mcm10 | N-terminal His tag |  | Ni-NTA Agarose  MonoS (twice) |  | 25 mM HEPES-KOH pH 7.6, 10% glycerol, 0.01% (v/v) NP-40-S, 1 mM EDTA, 1 mM DTT, 200 mM K-glutamate |
| ORC | CBP-cleavable tag on Orc1 |  | Calmodulin-Sepharose 4B  Superdex 200 |  | 25 mM HEPES-KOH pH 7.6, 0.05% NP-40 and 10% glycerol, 300 mM KOAc |
| PCNA | Untagged |  | Nucleic acid precipitation with Polymin P Ammonium sulfate precipitation  HiTrap SP HP (flow through)  HiTrap Heparin HP (flow through)  HiTrap DEAE Fast Flow  MonoQ  Superdex 200 |  | 25 mM Tris-HCl pH 7.2, 10% glycerol, 1 mM EDTA, 150 mM NaCl. |
| Pol α | N-terminal CBP tag on Pri1 | (Different storage buffer) | Calmodulin-Sepharose 4B  MonoQ  Superdex 200 |  | 25mM HEPES-KOH pH 7.6, 10% (v/v) glycerol, 400 mM KOAc, 0.02% (v/v) NP-40-S, 1mM DTT, 1 mM EDTA |
| Pol δ | C-terminal CBP tag on Pol32 |  | Calmodulin-Sepharose 4B  HiTrap Heparin HP  Superdex 200 |  | 25 mM Tris-HCl pH 7.2, 10% glycerol, 0.02% NP-40-S, 1 mM EDTA, 1 mM DTT, 150 mM NaCl |
| Pol ε | C-terminal CBP tag on Dpb4 |  | Calmodulin-Sepharose 4B  HiTrap Heparin HP  Superdex 200 |  | 25 mM HEPES-KOH pH 7.6, 10% glycerol, 1 mM DTT, 500 mM KOAc |
| RFC | N-terminal CBP tag on Rfc3 |  | Calmodulin-Sepharose 4B  MonoS  Superdex 200 |  | 25 mM HEPES-KOH pH 7.6, 10% glycerol, 1 mM DTT, 1 mM EDTA, 150 mM NaCl |
| RPA | Untagged | Devbhandari., 2017 | HiTrap Blue HP  ssDNA Cellulose  MonoQ |  | 25 mM Tris HCl pH 7.5, 1 mM EDTA, 10 % glycerol, 100 mM NaCl, 1 mM DTT |
| Sld2 | C-terminal 3xFLAG tag |  | Ammonium sulfate precipitation  Anti-FLAG M2 Agarose  HiTrap SP HP |  | 25 mM HEPES-KOH pH 7.6, 0.02% (v/v) NP-40-S, 1 mM EDTA, 1 mM DTT 40% (v/v) glycerol, 350 mM KCl |
| Sld3/7 | C-terminal cleavable TCP tag |  | IgG Sepharose Fast Flow  TEV removal with Ni-NTA Agarose  Superdex 200 |  | 25 mM HEPES-KOH pH 7.6, 10% glycerol, 0.02% (v/v) NP-40-S, 1 mM EDTA, 1 mM DTT, 500 mM KCl |
| TopoI | N-terminal cleavable CBP tag | (Different storage buffer) | Calmodulin-Sepharose 4B  TEV removal with Talon column  Superdex 200 |  | 25mM HEPES-KOH pH 7.6, 10% (v/v) glycerol, 400 mM KOAc, 0.02% (v/v) NP-40-S, 1mM DTT |
| Mrc1 | C-terminal 2xFLAG tag | Guilliam and Yeeles, 2020 | Anti-FLAG M2 Agarose  Superose 6 |  | 25 mM Tris-HCl pH 7.2, 10 % (v/v) glycerol, 0.02% (v/v) NP-40-S, 1mM DTT, 150 mM NaCl |
| Csm3-Tof1 | N-terminal CBP cleavable tag on Csm3 | Guilliam and Yeeles, 2020 | Calmodulin-Sepharose 4B  TEV removal  MonoQ  Superdex 200 |  | 25 mM Tris-HCl pH 7.2, 10 % (v/v) glycerol, 0.02% (v/v) NP-40-S, 1mM DTT, 150 mM NaCl |
| Pol η | N-terminal CBP tag | Guilliam and Yeeles, 2020 | Calmodulin-Sepharose 4B  HiTrap Heparin HP |  | 25mM HEPES-KOH pH 7.6, 10% (v/v) glycerol, 300 mM KOAc, 0.02% (v/v) NP-40-S, 1mM DTT |
| Rad6-Rad18 | N-terminal 2xFLAG tag on Rad18 | Guilliam and Yeeles, 2020 | Anti-FLAG M2 Agarose  HiTrap Heparin HP  MonoQ  Superdex 200 |  | 25mM HEPES-KOH pH 7.6, 10% (v/v) glycerol, 300 mM KOAc, 0.02% (v/v) NP-40-S, 1mM DTT |
| Uba1 | N-terminal 2xFLAG tag | Guilliam and Yeeles, 2020 | Anti-FLAG M2 Agarose  MonoQ |  | 25mM HEPES-KOH pH 7.6, 10% (v/v) glycerol, 300 mM KOAc, 0.02% (v/v) NP-40-S, 1mM DTT |
| Pol δ^cat^ | C-terminal CBP tag on Pol32 | Aria and Yeeles, 2019 | Ammonium sulfate precipitation  Calmodulin-Sepharose 4B  MonoQ |  | 25mM HEPES-KOH pH 7.6, 40% (v/v) glycerol, 300 mM KOAc, 0.02% (v/v) NP-40-S, 1mM DTT |
| Rev1 | N-terminal CBP tag | This study | Calmodulin-Sepharose 4B  HiTrap Heparin HP |  | 25mM HEPES-KOH pH 7.6, 10% (v/v) glycerol, 300 mM KOAc, 0.02% (v/v) NP-40-S, 1mM DTT |
| Pol ζ | N-terminal 2XFLAG tag on Rev3  C-terminal CBP tag on Pol32 | This study | Anti-FLAG M2 Agarose  Calmodulin-Sepharose 4B  HiTrap Heparin HP |  | 25mM HEPES-KOH pH 7.6, 10% (v/v) glycerol, 300 mM KOAc, 0.02% (v/v) NP-40-S, 1mM DTT |
| Pol ε^exo-^ | C-terminal CBP tag on Dpb4 | Same method as used for Pol ε^PIP^ (Aria and Yeeles, 2019) | Calmodulin-Sepharose 4B  Anti-FLAG M2 Agarose (flow through kept)  HiTrap Heparin HP  Superdex 200 |  | 25 mM HEPES-KOH pH 7.6, 10% glycerol, 1 mM DTT, 500 mM KOAc |

**Supplementary Table S5. Protocol references, tags, purification methods, and final buffer composition for purified proteins used in replication assays.**
