# Supplementary material for: The eukaryotic replisome tolerates leading‐strand base damage by replicase switching
Source: EMBO J. 2021 Feb 8;40(5):e107037. doi: 10.15252/embj.2020107037 (PMC7917549; doi:10.15252/embj.2020107037)
Supplement: Supplementary file 2 — Expanded View Figures PDF [file EMBJ-40-e107037-s002.pdf]

Expanded View Figures

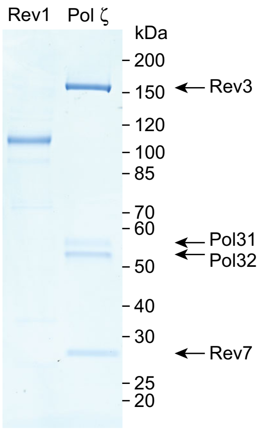

Figure EV1. (Related to Fig 1). Purified Rev1 and Pol ζ.

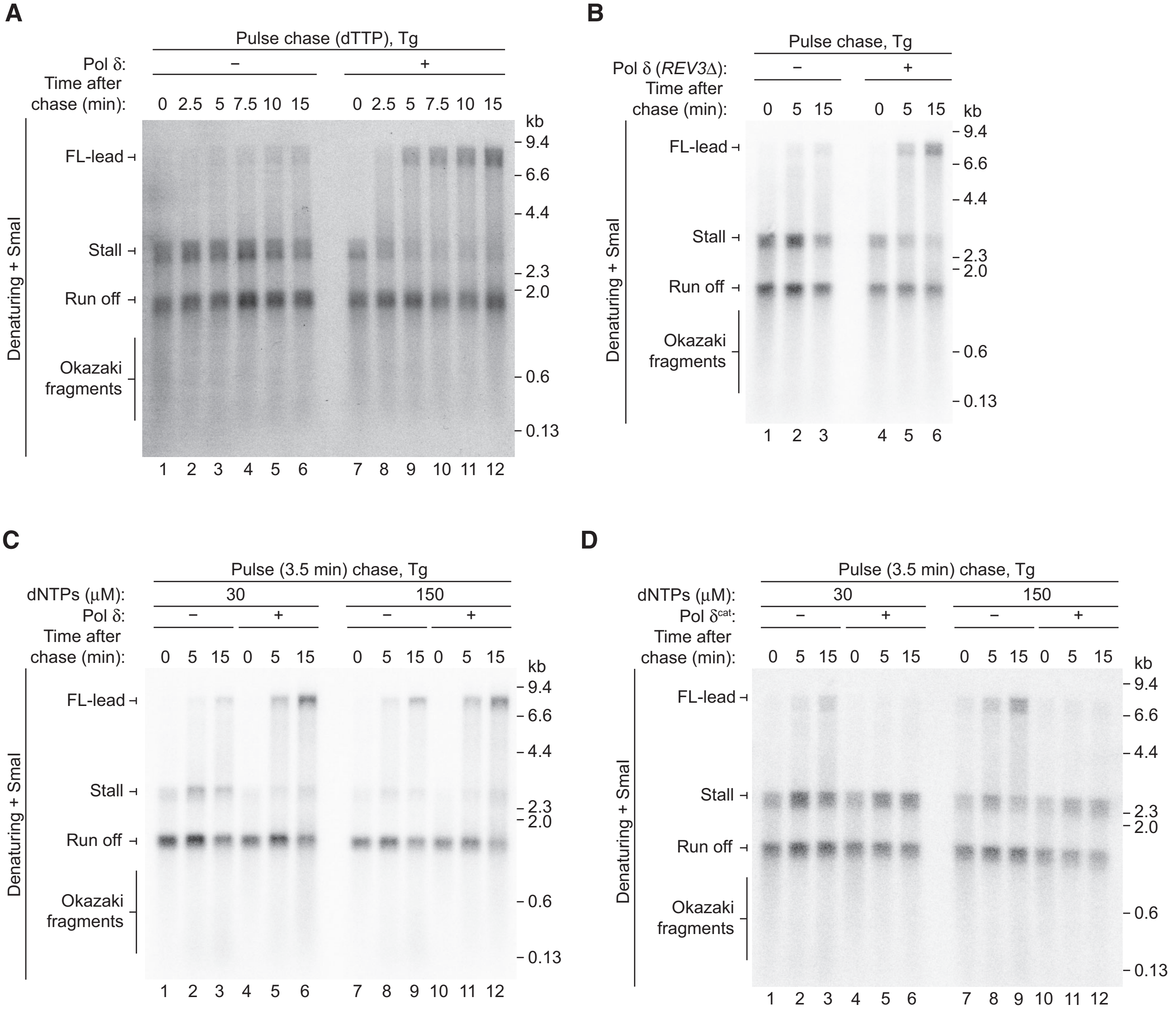

Figure EV2. (Related to Fig 3). Pol δ can bypass Tg.

A Pulse chase reaction as performed in Fig 3B but using ^{32}P-dTTP and elevated dTTP in the pulse and chase respectively.

B Pulse chase reaction in the absence and presence of Pol δ purified from the *REV3Δ* strain. The pulse phase of the reaction was 5 min.

C Pulse chase reaction at 30 and 150 μM dNTPs in the absence and presence of Pol δ. The chase was added 3.5 min after initiation of DNA synthesis. The dCTP concentration was 2.5 and 600 μM in the pulse and chase phases of the reactions, respectively.

D Pulse chase reaction as performed in (C) but using Pol δ^{cat} instead of Pol δ.

© 2021 The Authors

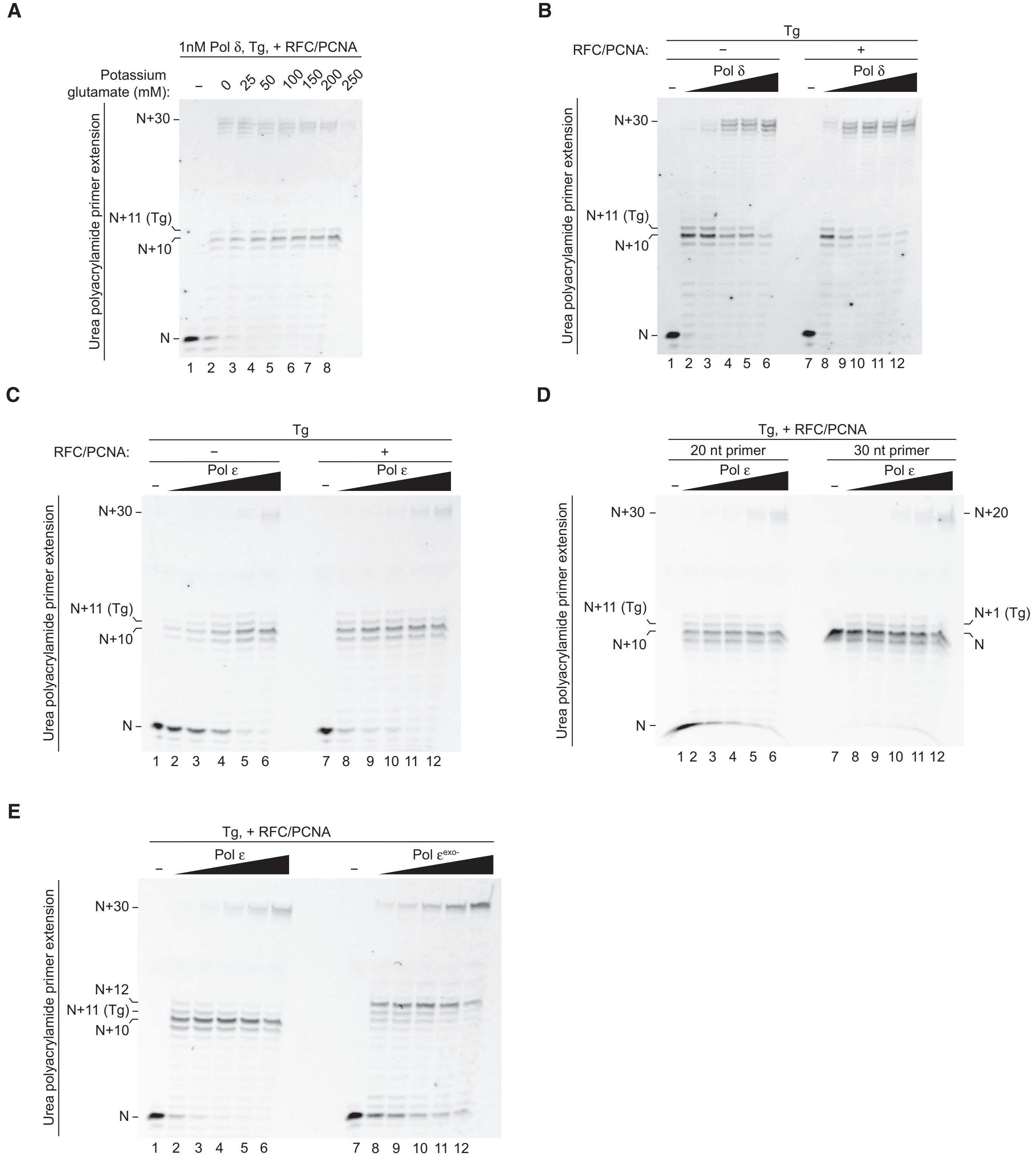
A
1nM Pol δ, Tg, + RFC/PCNA
Potassium glutamate (mM):
–
0
25
50
100
150
200
250
Urea polyacrylamide primer extension
N+30
N+11 (Tg)
N+10
N
1 2 3 4 5 6 7 8
B
Tg
RFC/PCNA:
–
+
Pol δ
Pol δ
N+30
N+11 (Tg)
N+10
N
1 2 3 4 5 6 7 8 9 10 11 12
C
Tg
RFC/PCNA:
–
+
Pol ε
Pol ε
N+30
N+11 (Tg)
N+10
N
1 2 3 4 5 6 7 8 9 10 11 12
D
Tg, + RFC/PCNA
20 nt primer
30 nt primer
Pol ε
Pol ε
N+30
N+11 (Tg)
N+10
N
N+20
N+1 (Tg)
N
1 2 3 4 5 6 7 8 9 10 11 12
E
Tg, + RFC/PCNA
Pol ε
Pol ε exo-
N+30
N+12
N+11 (Tg)
N+10
N
1 2 3 4 5 6 7 8 9 10 11 12

Figure EV3.

Figure EV3. (Related to Fig 3). Analysis of Tg bypass by Pol δ and Pol ε.

A Potassium glutamate titration into primer extension reactions on the Tg template shown in Fig 3G in the presence of Pol δ (1 nM), RFC, and PCNA.
B Pol δ titration (1, 2.5, 5, 7.5, 10 nM) into primer extension reactions on the Tg template shown in Fig 3G in the absence and presence of RFC and PCNA.
C Pol ε titration (0.5, 1, 2, 4, 8 nM) into primer extension reactions on the Tg template in the absence and presence of RFC and PCNA.
D Pol ε titration (0.5, 1, 2, 4, 8 nM) into primer extension reactions on the Tg template with either a 20 nt or 30 nt primer annealed in the presence of RFC and PCNA.
E Titration of Pol ε and Pol ε^{exo-} (1, 2, 4, 8, 16 nM) into primer extension reactions on the Tg template in the presence of RFC and PCNA.

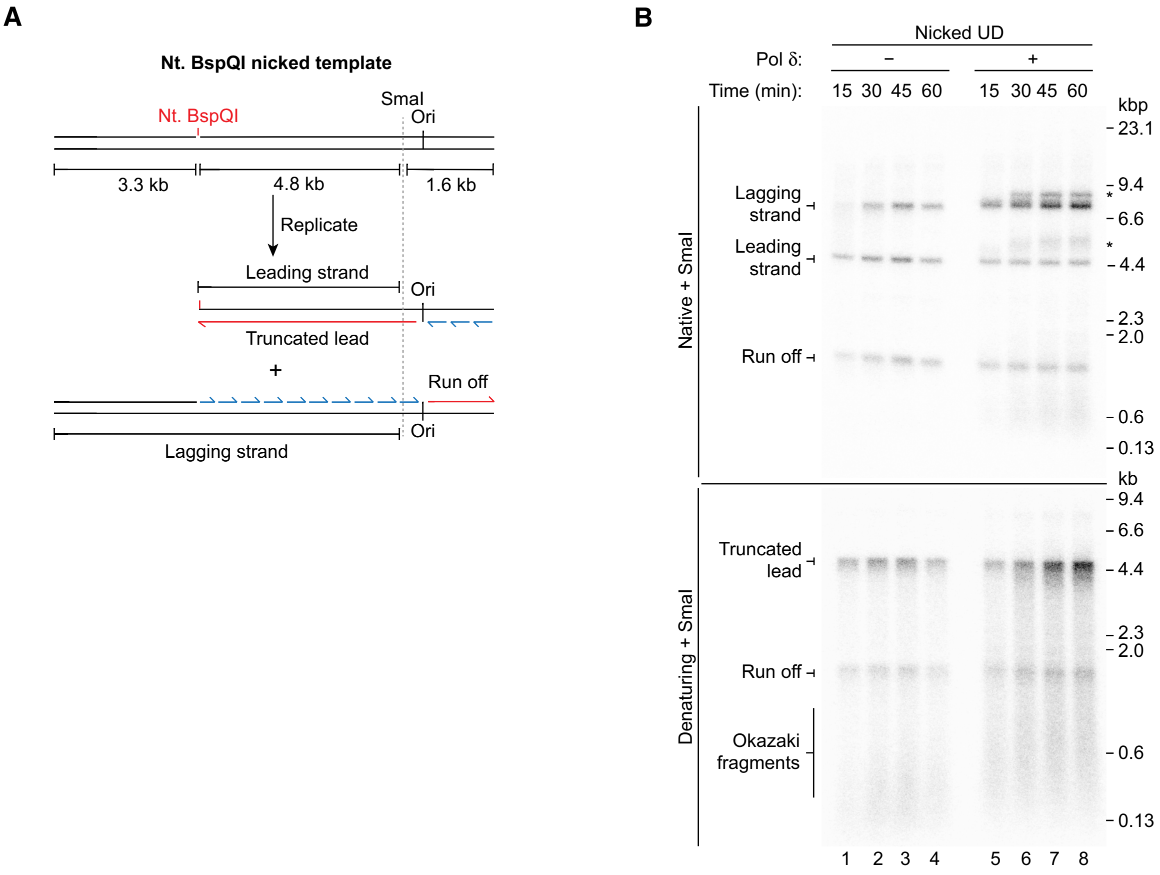

Figure EV4. (Related to Fig 5). Isolation of nascent leading strands.

A Schematic of the Nt. BspQI nicked UD template and reaction products generated in (B).
B Standard replication reaction on the Nt. BspQI nicked UD template performed in the absence and presence of Pol δ. *caused by Pol δ strand displacement activity.

© 2021 The Authors
